# *Giardia* and *Cryptosporidium* in resident wildlife species in Arctic Alaska

**DOI:** 10.1016/j.fawpar.2023.e00206

**Published:** 2023-08-29

**Authors:** Caroline Van Hemert, Lora R. Ballweber, David R. Sinnett, Todd C. Atwood, Anthony Fischbach, David D. Gustine, Kristy L. Pabilonia

**Affiliations:** a U.S. Geological Survey Alaska Science Center, Anchorage, AK, USA; b Colorado State University Veterinary Diagnostic Laboratories, Fort Collins, CO, USA; c U.S. Department of Agriculture APHIS Wildlife Services, Palmer, AK, USA; d Current affiliation: U.S. Fish and Wildlife Service, Marine Mammals Management—Polar Bears, Anchorage, AK, USA

**Keywords:** Arctic fox, Caribou, *Cryptosporidium*, *Giardia*, Polar bear, Pacific walrus

## Abstract

*Giardia* and *Cryptosporidium* are zoonotic protozoan parasites that can infect humans and other taxa, including wildlife, often causing gastrointestinal illness. Both have been identified as One Health priorities in the Arctic, where climate change is expected to influence the distribution of many wildlife and zoonotic diseases, but little is known about their prevalence in local wildlife. To help fill information gaps, we collected fecal samples from four wildlife species that occur seasonally on the northern Alaska coastline or in nearshore marine waters—Arctic fox (*Vulpes lagopus*), polar bear (*Ursus maritimus*), Pacific walrus (*Odobenus rosmarus divergens*), and caribou (*Rangifer tarandus*)—and used immunofluorescence assays to screen for *Giardia* cysts and *Cryptosporidium* oocysts. We detected *Giardia* cysts in 18.3% and *Cryptosporidium* oocysts in 16.5% of Arctic foxes (*n* = 109), suggesting that foxes may be potentially important hosts in this region. We also detected *Giardia* cysts in a single polar bear (12.5%; *n* = 8), which to our knowledge represents the first such report for this species. Neither parasite was detected in walruses or caribou.

## Highlights

• We surveyed for *Giardia* spp. and *Cryptosporidium* spp. in wildlife from Arctic Alaska.
• Arctic foxes are important hosts for both parasites in this region.
• Detection of *Giardia* in a single polar bear represents the first such report for this species.
• We provide new information about two parasites of high zoonotic concern in the Arctic.

## 1 Introduction

The Arctic is undergoing rapid environmental change, with current estimates of warming more than four times the global rate (Rantanen et al., 2022). Among other climate-related challenges, certain wildlife and zoonotic diseases, particularly those with environmental transmission cycles, are expected to increase or expand (Davidson et al., 2011; Jenkins et al., 2013). However, baseline knowledge about current distribution patterns is lacking for Arctic regions, making risk assessments difficult and projections of future impacts largely speculative.

*Giardia* and *Cryptosporidium* are zoonotic protozoan parasites that can cause gastrointestinal disease, specifically diarrhea, in humans and animals. These parasites have global distribution and are primarily transmitted through fecal-oral pathways, often by way of contaminated water or food sources (Robertson and Debenham, 2022). Previous surveys of Arctic wildlife suggest that *Giardia* is well established in some terrestrial and marine species and that *Cryptosporidium* is also present, although local transmission of the latter has been less evident (Robertson and Debenham, 2022). Both parasites have been identified as potentially sensitive to climate warming, including in northern regions (Finlayson-Trick et al., 2021; Robertson and Debenham, 2022).

Because *Giardia* and *Cryptosporidium* can be considered in both zoonotic (from wildlife to animals) and anthroponotic (from humans to animals) contexts, understanding more about current distribution patterns and potential routes of transmission among wildlife is important for human, domestic animal, and wildlife health, thus making them priorities within a One Health framework (Jenkins et al., 2015). Enteric disease is a major health concern in rural and remote communities in the Arctic (Finlayson-Trick et al., 2021; Jenkins et al., 2013), where subsistence activities are prevalent, and a recent Centers for Disease Control and Prevention One Health zoonotic disease prioritization workshop for Alaska identified *Giardia* and *Cryptosporidium* as pathogens of high concern (https://www.cdc.gov/onehealth/pdfs/Alaska-508.pdf; accessed on 2023-01-05). Human residents of Alaska and northern Canada have high rates of giardiasis (Jenkins et al., 2013; Mosites et al., 2018) and are also frequently exposed to *Cryptosporidium* (Finlayson-Trick et al., 2021; Mosites et al., 2018).

Like other waterborne parasites, transmission of *Giardia* and *Cryptosporidium* may be affected by climate-related changes in hydrologic cycles, such as increased rates of precipitation, permafrost thaw, and coastal flooding associated with extreme weather events, while rising temperatures allow for longer persistence of cysts and oocysts in the environment (Jenkins et al., 2013). Shifts in wildlife movements could also contribute to the spread of these parasites, with wildlife playing a role in disbursement or serving as novel hosts (Davidson et al., 2011). For instance, northward range expansion from subarctic to Arctic environments has been documented among a variety of migratory and resident species, including beavers (Tape et al., 2022), which are thought to serve as reservoirs of *Giardia* (Tsui et al., 2018). At the same time, encounters between marine and terrestrial wildlife are increasing due to the reduction of nearshore sea ice, potentially creating new opportunities for pathogen exchange among species with limited historical contact (Post et al., 2013).

Multiple genotypes of *Giardia* and *Cryptosporidium* exist; some of these are known to be zoonotic, whereas others are considered taxon-specific with limited potential to cause human illness (Ryan and Zahedi, 2019). Both wild and domestic animals are thought to be possible reservoirs of zoonotic strains but the actual contribution of wildlife to human infections remains largely unresolved (Kutz et al., 2009; Robertson and Debenham, 2022). Conversely, many animal species are susceptible to human-derived strains of *Giardia* and *Cryptosporidium* and several studies have identified apparent parasite spillover from human populations to Arctic wildlife (Kutz et al., 2008; Thompson, 2013). Although the health impacts of *Giardia* and *Cryptosporidium* have not been well studied in wildlife, diarrheal illness from these parasites has been reported among cats, dogs, sheep, and other domestic mammals (Ryan and Zahedi, 2019; Thompson et al., 2008). Infectious disease spillover events can negatively impact free-ranging populations and even sublethal parasite infections sometimes result in long-term demographic declines (Thompson et al., 2009).

In Alaska, few wildlife surveys for *Giardia* and *Cryptosporidium* have been reported in the past decade; of these, none include samples collected from the Arctic coast, where significant ecological changes are currently underway (Post et al., 2013). Identifying wildlife species that may serve as possible reservoirs or, alternatively, as sentinels of parasite exposure will help guide further research and surveillance efforts and inform One Health needs in the region.

In this study, we tested opportunistically collected fecal samples from four Arctic wildlife species that occur seasonally on the northern Alaskan coast or in nearshore marine waters: Arctic fox (*Vulpes lagopus*), polar bear (*Ursus maritimus*), Pacific walrus (*Odobenus rosmarus divergens*), and caribou (*Rangifer tarandus*). Our primary objective was to estimate the prevalence of *Giardia* and *Cryptosporidium* in these species, for which limited background information exists.

## 2 Methods

We collected feces immediately (<5 min) after death from Arctic foxes (*n* = 109) that were shot or trapped near Utqiaġvik (formerly Barrow), Alaska (71° 17′ N, 156° 47′ W) during May and June 2014 and 2016 as part of an ongoing U.S. Fish and Wildlife Service predator control program managed by the U.S. Department of Agriculture. Fox feces were collected when spontaneously voided or via fecal grab. We captured polar bears during March and April 2015 on the sea ice of the southern Beaufort Sea between Utqiaġvik and the United States-Canada border as described in Atwood et al. (2017) and collected feces by fecal grab from the rectum of chemically immobilized bears (*n* = 31; U.S. Geological Survey Alaska Science Center ACUC # 2010–14). We captured caribou on their wintering grounds in the east-central Brooks Range (67° 1′–67° 50′ N, 147° 45′–149° 0′ W) in April 2015 and 2017 as described in Johnson et al. (2020) and obtained samples from individuals (*n* = 55) when feces were spontaneously voided or via digital palpation (U.S. Geological Survey Alaska Science Center ACUC #2015–5). We collected walrus fecal samples (*n* = 61) from sea ice pans in May–July of 2012–2015 in the eastern Chukchi Sea (70° 41′–71° 32′ N, 161° 22′–166 16′ W; USFWS scientific research permit MA801652–8). When multiple walrus fecal samples were present on a single ice pan, we limited collection to samples that were separated by >3 m (equivalent to a full walrus body length). After collection and transport (typically <1 h) all fecal samples were stored at −20 °C and remained frozen until analysis.

We analyzed fecal samples for *Giardia* and *Cryptosporidium* by immunofluorescence assay (IFA) at the Colorado State University Veterinary Diagnostic Laboratories with previously established methods (Hughes-Hanks et al., 2005). Briefly, we used the MERIFLUOR *Cryptosporidium/Giardia* direct immunofluorescent assay (Meridian Bioscience Inc., Cincinnati, Ohio) according to the manufacturer's directions, with samples run in batches with positive and negative controls. We examined slides at 100×, and if cysts or oocysts were not detected, then also at 200× magnification using a fluorescence microscope. We considered a sample to be positive if any *Giardia* cysts *or Cryptosporidium* oocysts were detected. A subset of polar bear samples (*n* = 23) had inadequate volume for IFA and were therefore excluded from analysis.

Genomic DNA was extracted from fecal samples that were positive for *Giardia* or *Cryptosporidium* by IFA following an established protocol (Da Silva et al., 1999) and stored at −20 °C until subsequent PCR assays. We performed polymerase chain reaction (PCR) assays targeting *Giardia* glutamate dehydrogenase (gdh), beta-giardin (bg), and triosephosphate isomerase (tpi) genes and *Cryptosporidium* heat-shocked protein (hsp70), and small subunit ribosomal RNA (SSU-rRNA) for molecular identification of the respective pathogens. Previously described PCR protocols were used (Cacciò et al., 2002; Morgan et al., 1997; Morgan et al., 2001; Read et al., 2004; Sulaiman et al., 2003; Xiao et al., 1999) with the following modifications: all PCR reactions were performed in 25-μl reactions using HotStarTaq Master Mix (Qiagen, Valencia, CA) with 1.5 mM of MgCl_2_, and 1 (*Cryptosporidium*) or 2 (*Giardia*) μl of template DNA. Validated positive and negative controls were included in each run.

We used Epitools with Wilson's estimate (Ausvet Pty Ltd., Canberra, Australia; https://epitools.ausvet.com.au) to calculate prevalence and 95% confidence intervals (CI) for parasite prevalence.

## 3 Results

We detected *Giardia* in 18.3% (95% CI: 12.2–26.6%) and *Cryptosporidium* in 16.5% (95% CI: 10.7–24.6%) of Arctic foxes (*n* = 109; Table 1). Eight foxes (7.3%) were positive for both parasites. We also detected *Giardia* in a single polar bear sample (12.5%; 95% CI: 2.2–47.1%, *n* = 8). All caribou (*n* = 55) and walrus (*n* = 61) samples were negative for both *Giardia* and *Cryptosporidium* (Table 1; Van Hemert, 2023). Amplification of positive fox and polar bear samples was unsuccessful and therefore further molecular analyses of *Giardia or Cryptosporidium* parasites was not possible. Poor amplification of *Giardia* and other parasite DNA has been reported in previous studies of canids, specifically foxes, and it has been suggested that PCR inhibitors may be present in the fecal matrix of canids, potentially explaining in part our lack of molecular detections (Robertson et al., 2019). Negative results, while not informative for genotyping, can help identify challenges with the use of traditional PCR methods to identify parasites in foxes.

**Table 1:** Detections of *Giardia* cysts and *Cryptosporidium* oocysts in fecal samples from four resident wildlife species (Arctic fox [*Vulpes lagopus*], polar bear [*Ursus maritimus*], Pacific walrus [*Odobenus rosmarus divergens*], and caribou [*Rangifer tarandus*]) collected in Arctic Alaska.

			*Giardia*		*Cryptosporidium*	
	*n*	Year(s)	No. +	Prevalence (95% CI)	No. +	Prevalence (95% CI)
Arctic fox	109	2014, 2016	20	18.3 (12.2–26.6)	18	16.5 (10.7–24.6)
Polar bear	8	2015	1	12.5 (2.2–47.1)	0	–
Pacific walrus	61	2012–2015	0	–	0	–
Caribou	55	2015, 2017	0	–	0	–

## 4 Discussion

This study provides new information about the occurrence of two parasites of high zoonotic concern in wildlife from Arctic Alaska. We report *Giardia* in polar bears and identify Arctic foxes as potentially important hosts of both *Giardia* and *Cryptosporidium* in this region. The lack of detection of these parasites in caribou and walrus suggests that prevalence is currently low and provides a baseline for future studies in the context of a rapidly changing environment.

**Fig. 1:**
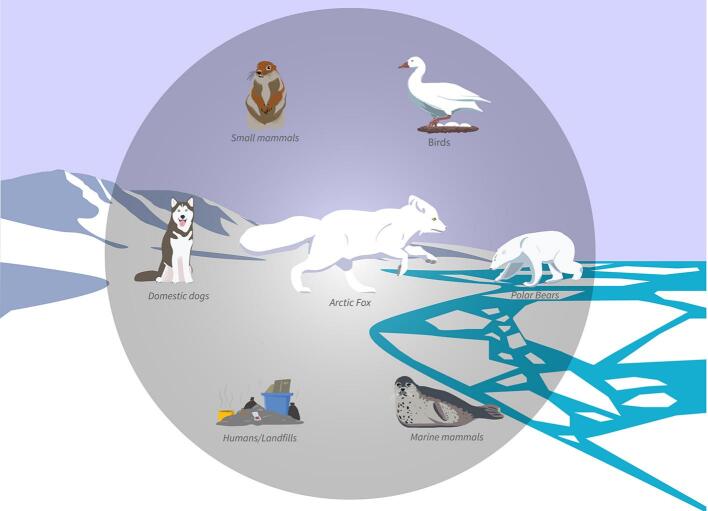
Conceptual model illustrating potential contact opportunities between Arctic foxes (*Vulpes lagopus*), domestic animals, humans, and other wildlife species in the Alaskan Arctic. We detected *Giardia* cysts and *Cryptosporidium* oocysts in 18.3 and 16.5% of foxes, respectively, suggesting that they may play an important role in parasite ecology in this region. Illustration by Toshio Matsuoka, U.S. Geological Survey.

Our results suggest that foxes may play an important role in parasite ecology in northern Alaska, consistent with a study of Arctic foxes in the central Canadian Arctic, which reported *Giardia* in 16% and *Cryptosporidium* in 8% of foxes (Elmore et al., 2013). In contrast, neither parasite was detected in Arctic foxes from Svalbard, Norway (Myšková et al., 2019). Although all three of these Arctic regions are sparsely populated, Svalbard is the most northerly and, as a remote archipelago, has strict animal import regulations, which may limit exposure of wildlife to parasites of domestic animal origin. Elsewhere, including in other parts of Europe, *Giardia* and *Cryptosporidium* have been commonly detected in both wild and domestic canids, including red foxes (*Vulpes vulpes*) (Kváč et al., 2021; Robertson et al., 2019). Like red foxes, Arctic foxes are opportunistic predators and scavengers and, as a result, engage in many inter-specific interactions (Chesemore, 1968; Samelius and Alisauskas, 2000) (Fig. 1). Throughout the year, they feed on microtine rodents, ground squirrels, and other small mammals (Fuglei and Ims, 2008; Savory et al., 2014), some of which are known to be reservoirs of multiple enteric parasites, including *Giardia* and *Cryptosporidium* (Appelbee et al., 2005; Ryan and Zahedi, 2019). They also consume waterfowl (Samelius and Alisauskas, 2000), which can serve as parasite vectors over long distances (Ryan and Zahedi, 2019). In winter, some Arctic foxes follow polar bears onto the sea ice to scavenge marine mammal carcasses or enter subnivean lairs to hunt seals pups (Pamperin et al., 2008; Roth, 2002). Arctic foxes also readily exploit anthropogenic food resources in and around human communities (Savory et al., 2014) and may therefore be exposed to food or water sources contaminated by untreated human sewage or feces of domestic animals (Fig. 1). It is currently unclear what health effects, if any, infection with *Giardia* or *Cryptosporidium* may have on Arctic foxes, but other canids have been reported with diarrhea and weight loss in clinical settings (Thompson et al., 2008). Like many Arctic wildlife species, Arctic foxes face various threats due to climate change, including interspecific competition from red foxes undergoing northward range expansion (Fuglei and Ims, 2008) and changing pathogen and parasite pressures (Davidson et al., 2011; Robertson and Debenham, 2022).

Without molecular data we cannot assess likely sources of *Giardia* or *Cryptosporidium* infections in Arctic fox, but previous reports from red foxes suggest a possible role in zoonotic transmission cycles (Barrera et al., 2020; Robertson et al., 2019). The majority of *Giardia* DNA previously characterized from red fox samples has consisted of Assemblages A and B, both of which are typically associated with humans, indicating that foxes may serve as potential wildlife reservoirs while also being subject to human-origin spillover events (Robertson et al., 2019). However, amplification of *Giardia* isolates from positive fox samples has had a relatively poor success rate and the relationship between infections in wild foxes and in humans remains inconclusive (Robertson et al., 2019). Targeted method development is warranted to improve success of amplification and molecular identification of *Giardia* among foxes and other canids. Like *Giardia*, genotypes of *Cryptosporidium* typically associated with humans and domestic animals have also been detected in red foxes (Barrera et al., 2020; Mateo et al., 2017). Most of the Arctic fox samples from our study were collected within 40 km of Utqiaġvik, a community of about 5000 residents, and anthropogenic spillover is plausible. Domestic dogs are common in the Alaskan Arctic and may also be a source of zoonotic or canid-specific parasites, as has been reported in northern Canada (Himsworth et al., 2010; Julien et al., 2019; Salb et al., 2008). Additional efforts to amplify and sequence positive samples is necessary to help clarify transmission pathways of *Giardia* and *Cryptosporidium* in Arctic foxes in Alaska, including possible zoonotic and/or anthroponotic cycles.

To our knowledge, the detection of *Giardia* in a single polar bear from the southern Beaufort Sea is the first such report for this species. Given the small sample size (*n* = 8), we cannot infer prevalence of *Giardia* across the larger population, but this finding indicates that additional monitoring of polar bears is warranted (Table 1). Sparse reports of *Giardia* in bears currently exist (Aghazadeh et al., 2015; Figueroa, 2015; Roach et al., 1993; Ryan and Zahedi, 2019); of these, clinical presentation of giardiasis has not been reported, although most contemporary wildlife health programs are not sufficiently robust to evaluate sublethal impacts from pathogens, including diarrheal disease. Given their vulnerability to additional climate-related stressors related to diminishing sea ice, expanding transportation corridors, and other anthropogenic activities in the Arctic, polar bear health is of high conservation concern (Atwood et al., 2017). We cannot determine the source of the single *Giardia* infection we identified but multiple pathways are plausible. *Giardia* parasites have been detected in ringed (*Pusa hispida*) (Hughes-Hanks et al., 2005) and bearded seals (*Erignathus barbatus*) (Dixon et al., 2008), two important prey species for polar bears (Rode et al., 2022), as well as bowhead whales (*Balaena mysticetus*) (Hughes-Hanks et al., 2005), which polar bears commonly scavenge on shore after community harvests in Alaska (Wilson et al., 2017). As polar bears spend increasing amounts of time on land, they may be more frequently exposed to pathogens of terrestrial and/or anthropogenic origin, a pattern that has been observed in the southern Beaufort Sea and among other polar bear populations (Atwood et al., 2017; Pilfold et al., 2021; Smith et al., 2022). Further surveillance for protozoan parasites is needed to determine whether *Giardia* or *Cryptosporidium* pose potential health risks to polar bears.

We did not detect *Giardia* or *Cryptosporidium* in the 55 caribou samples we examined (Table 1), indicating relatively low prevalence in our study areas. To date, *Giardia* has not been reported in ungulates from the Alaskan Arctic but was identified in muskoxen from Banks Island (Kutz et al., 2008) and caribou from the Northwest Territories (Johnson et al., 2010) of the Canadian Arctic. In the case of muskoxen surveyed on Banks Island, *Giardia* isolates were identified as belonging to Assemblage A (*Giardia duodenalis*), a human-origin strain (Kutz et al., 2008), implicating likely spillover of *Giardia* from humans to wildlife. *Cryptosporidium* was previously reported in 6% of caribou from the Western Arctic and Teshekpuk Lake herds (Siefker et al., 2002), whose annual range overlaps the Central Arctic Herd from which our samples were collected (Prichard et al., 2020). Genotyping of a single *Cryptosporidium* isolate from the earlier study identified a novel lineage (Siefker et al., 2002), but follow up work has not been conducted since that time.

We also did not detect *Giardia* or *Cryptosporidium* in walruses (Table 1), contrary to studies of other marine mammals in the North American Arctic, including among phocids as well as toothed and baleen whales (Dixon et al., 2008; Hughes-Hanks et al., 2005). We are unaware of prior surveys of walruses for these parasites and our data provide a useful baseline for a species that may be vulnerable to future exposure. Walrus diets consist primarily of benthic invertebrates, particularly clams and other large-bodied invertebrates (Sonsthagen et al., 2020), which can concentrate *Giardia* cysts and *Cryptosporidium* oocysts (Robertson, 2007). In the Canadian Arctic, *Giardia* and *Cryptosporidium* have been detected in locally-harvested clams and mussels (Finlayson-Trick et al., 2021), demonstrating that these taxa could serve as vectors of parasite infection to wildlife and human consumers. Additionally, some seasonal walrus haul-outs occur near human communities, where environmental contamination from anthropogenic sources is possible (Fischbach et al., 2022). Given the presence of *Giardia* and *Cryptosporidium* in species that co-occur with walruses and a plausible dietary source of exposure, continued monitoring of this species is warranted. Complementary sampling of benthic invertebrates, including clams, would also help evaluate the possibility that they may serve as local parasite vectors.

Although sample sizes were limited, this report offers preliminary insights into the occurrence of two parasites of high zoonotic concern in wildlife from Arctic Alaska. We report detection of *Giardia* in a polar bear and identify Arctic foxes as potentially important hosts of both *Giardia* and *Cryptosporidium* in this region. The lack of detection of these parasites in caribou and walrus, while not definitive proof of their absence, suggests that prevalence is currently low and provides a baseline for future studies in the context of a rapidly changing environment. Future molecular work to identify genotypes/assemblages of *Giardia* and *Cryptosporidium* would provide important information about routes of transmission and the potential role of wildlife as zoonotic reservoirs or, conversely, their vulnerability to spillover events. Wildlife exposure to pathogens of anthropogenic origin is expected to increase in the Arctic as new transportation corridors and other infrastructure are developed in response to warming, and disease may present a compounding stressor for some populations (Smith and Stephenson, 2013). Additional sampling of wildlife and domestic animals (particularly dogs) throughout northern Alaska would help determine whether these parasites currently have widespread distribution across the Alaskan Arctic and if their occurrence is linked to specific environmental factors or proximity to human communities. Surveillance among human residents, in accordance with public health needs and community interests, could also inform existing One Health concerns in the context of a rapidly changing Arctic (Finlayson-Trick et al., 2021).

## Authors' contributions

CV conceived the study, interpreted data, and wrote the manuscript. LB conducted *Giardia* and *Cryptosporidium* analyses and contributed substantively to manuscript writing. DS contributed to study design and sample acquisition and managed the Arctic fox project. TA, AF, and DG oversaw other wildlife sampling programs and contributed substantively to manuscript writing. KP helped conceive of the study and managed laboratory procedures. All authors read and approved the final manuscript.

## Declaration of Competing Interest

The authors declare that they have no known competing financial interests or personal relationships that could have appeared to influence the work reported in this paper.
